# Is Leukocytosis a Predictor for Recurrence of Ischemic Events after Coronary Artery Bypass Graft Surgery? A Cohort Study

**DOI:** 10.5402/2012/824730

**Published:** 2012-07-03

**Authors:** Farid Rashidi, Peiman Jamshidi, Marziah Kheiri, Shadi Ashrafizadeh, Amir Ashrafizadeh, Fatemeh Abdolalian, Fatemeh Mirzamohamadi

**Affiliations:** ^1^Shahid Madani Heart Center, Tabriz University of Medical Sciences, Tabriz, Iran; ^2^Swiss Cardiovascular Center, Bern University Hospital, Bern, Switzerland; ^3^Azad University of Medical Sciences, Tabriz, Iran; ^4^Northwest Health, NSW Australia, Tamworth, Australia; ^5^Valiasr Hospital, Zanjan University of Medical Sciences, Zanjan, Iran

## Abstract

*Objective*. Studies have shown that inflammation plays an important role in pathogenesis of coronary artery disease. The present study was designed to evaluate the role of high WBC count before CABG in predicting the risk of ischemic events after CABG. *Methods and Results*. This prospective study was carried out on 380 patients who underwent CABG surgery. Ninety seven patients (25.5%) had recurrent ischemic event. Mean WBC count before CABG surgery in patients with recurrent ischemic event was 7267 mic/lit ± 1863, which was significantly higher than the others, with a mean WBC count of 6721 mic/lit ± 1734 (*P* = 0.011). Patients with a WBC count more than 6000 mic/lit were at the highest risk for recurrent ischemic event (OR = 2.11, 95% CI = 1.18–3.44, *P* = 0.009). After adjustment for age, sex, family history, smoking, hyperlipidemia, Logestic Euro score, post opretive enzyme release (CK.mb), arterial graft and BMI, the relationship between the group with WBC count higher than 6000 mic/lit and recurrent of ischemic event remained significant (OR = 2.25, 95% CI = 1.2 to 4, *P* = 0.005). *Conclusions*. High WBC count before CABG surgery is an independent risk factor for ischemic events one year after the surgery.

## 1. Introduction

Inflammation plays a significant role in progression and pathogenesis of coronary artery disease [1]. Several studies have shown an association between high white blood cell (WBC) count and progression of coronary artery disease (CAD) [2, 3]. Leukocytosis also increases long-term mortality in patients with CAD. For the first time, the association between leukocytosis and CAD was described by Friedman et al. [2] Various factors affect the prognosis of coronary artery bypass graft (CABG) surgery [4, 5]. Inflammatory factors such as C reactive protein (CRP) level before CABG has been described as one of these factors [6]. Limited studies has suggested leukocytosis as one of the prognostic factors for in-hospital mortality after CABG [7]. Leukocytosis may also increase mortality following CABG surgery [8]. However, no study has investigated the role of high WBC count in increasing the risk of recurrent ischemic event after CABG surgery. In this study we investigated the role of WBC count before surgery in predicting the risk of recurrent ischemia after surgery.

## 2. Method and Materials

The study was carried out from October 2004 to January 2007 and the study protocol was approved by the ethics committee of Tabriz University of Medical Sciences. Studied population included all patients who were hospitalized in Shahid Madani Heart Center in Tabriz, Iran for elective CABG surgery. After signing a consent form, all patients were interviewed, using a structured questionnaire, by a trained staff physician. Collected data included age, gender, anthropometrics data, history of hypertension, diabetes, smoking, cardiac surgery, chronic obstructive pulmonary disease, ejection fraction prior to the surgery and the number of significantly involved arteries in angiography. Surgical data including the surgical method (on pump or off pump), number of grafts, duration of pump attachment, and duration of the surgery were recorded after the surgery.

Patients older than 70 years old or patients with a history of MI in the past three months, history of infection, trauma, gastrointestinal bleeding or surgery a week before the surgery, history of malignancy in the past three years, and an ejection fraction less than 30% were excluded. Out of 422 patients, 42 patients meet the exclusion criteria and a total of 380 patients were included in the study and were followed for one year.

After obtaining written informed consent, blood sample of each included patients was drawn and sent to laboratory for complete blood count (CBC), cholesterol, triglyceride (TG), low-density lipoprotein (LDL), high-density lipoprotein (HDL), blood urea (BUN), and creatinin (Cr) levels in standard situation.

## 3. Followup

Patients were followed for one year after the surgery. In-hospital followup included obtaining information regarding in-hospital mortality and postsurgical complications such as any need for intra-aortic balloon pump, ventricular fibrillation (VF), atrial fibrillation (AF), ischemic symptoms, severe post surgical bleeding, acute myocardial infarction (MI), infection and incidence of neurological complications during post-surgical hospital course. One year after the surgery, patient or the family (in case of patient's death) was contacted with phone or in person. To prevent bias, patients were followed by the same group of investigators who had interviewed the patient initially and were also blind to the result of the laboratory tests.

In followup contact, the patients were investigated regarding the overall mortality and morbidity due to cardiac causes, incidence of recurrent ischemic events or new onset cerebrovascular accident (CVA). Recurrent of ischemic event was defined as a new onset MI, unstable angina leading to hospitalization, stable angina, or revascularization (PTCA or CABG) after the initial surgery. Stable angina was defined as the occurrence of typical chest pain. Chest pain was considered to be typical when any of the following criteria were met: (1) pain in exercise, (2) retrosternal chest pain, or (3) remedy with resting or taking sublingual TNG. Mortality from cardiac origin was defined as sudden cardiac death or death in hospital following hospitalization because of MI or unstable angina (U/A). Patients who were hospitalized because of resting chest pain or chest pain with minimum exercise included in the U/A category. In addition, patients who met any two of the following three criteria were regarded to have MI: (1) chest pain longer than 30 minutes, (2) ST segment elevation, and (3) elevated cardiac enzymes. 

## 4. Statistic Analysis

SPSS 11.5 statistical software package (SPSS Inc., Chicago, IL) was used in statistical analysis. Statistical significance was set at *P* ≤ 0.05. The distribution of age, sex, laboratory values, and body mass index (BMI), were analyzed by means and standard deviation for categorical variables. Chi-square test was used to find whether patients who had recurrent ischemic events were different in regards to the presence of leukocytosis before surgery.

To estimate and adjust for confounding variable, multivariable stepwise forward and backward logistic regression tests were used. For multivariate analysis, we entered all risk factor for recurrence of ischemic events and all covariates with *P* < 0.05 on univariate analysis. Student *t*-test was used to compare the mean of WBC count, age, BMI, and ejection fraction in patients with or without recurrent event. 

## 5. Results

After applying inclusion and exclusion criteria, 380 patients were included in statistical analysis. Two hundred eighty one patients (73.9%) were male and 99 (26.1%) were female. Mean age of the patients was 57 years old (Table 1). Overall, 97 (25.5%) patients had recurrent ischemic event. The difference between the mean WBC count in patients with and without a recurrent ischemic event was significant (7267 mic/lit ±1863 versus 6721 mic/lit ±1734) (*P* = 0.011). Thirty days followup showed that the patients with WBC count more than 6000 mic/lit had higher risk for atrial fibrillation (OR = 2.38, 95% CI = 1.1–5.1, *P* = 0.021) (Table 2). Patients with a WBC count more than 6000 mic/lit were at the highest risk for recurrent of ischemic event (OR = 2.11, 95% CI = 1.18–3.42, *P* = 0.009) (Table 3). Sensitivity of WBC count higher than 6000 mic/lit for prediction of recurrent ischemia was 77% (95% CI = 67–84) (Table 4). There was not any difference between the patients with and without recurrent ischemic events in regards to other risk factors such as diabetes mellitus (DM), hypertension (HTN), family history, hypercholesterolemia, renal failure and smoking (Table 5). After adjustment for age, sex, family history, smoking, hyperlipidemia, Logistic Euro score, postoperative enzyme release (CK.mb), arterial graft, and BMI, the relationship between the group with WBC count higher than 6000 mic/lit and recurrent of ischemic event remained significant (OR = 2.25, 95%  CI = 1.2 to 4, *P* = 0.005) (Table 6).

There was not any difference between the two group in regards to medications such as ASA, Beta blocker, ACE.Inh, Statin, CCB, Nitrate and Diuretic (Table 5).

Two hundred and five patients (53.9%) were operated with off pump method and 175 (46.1%) patients with on pump method. The mean pump timing was 99.7 ± 30 minute and the mean surgery time was 245 minute. There was not any difference in recurrent ischemic events in patient with “on-pump” and “off-pump” method, (*P* = 0.53). The difference of pump timing between the patients with and without recurrent events was not significant (100.07/min versus 99.5/min, *P* = 0.59). Overall, the mean graft number was 2.5. Patients with and without recurrent events did not have significant difference in regard to the graft number (*P* = 0.38). 

## 6. Discussion

This study shows that elevated WBC count, after elective CABG surgery, is an independent risk factor for recurrent ischemic event one year after the surgery. The significance of elevated WBC, as a risk factor for recurrent ischemic event, stayed the same in multivariate analysis. To the best of our knowledge, our study is the only study which evaluated the effect of elevated WBC count after CABG and the risk of ischemic event one year after the surgery. Because of the relatively small sample size, we were not able to have a precise evaluation of elevated WBC and one-year mortality. 

Our study is well in accordance to the others and supports the role of inflammatory factors in prognosis of coronary artery disease. The role of elevated WBC count and coronary artery disease risk had been discussed previously [9, 10]. Our result is also in accordance with the findings of others which have showed the effect of elevated WBC count before CABG on postoperative mortality and early and late complications [7, 11, 12]. The relationship between increased WBC count and early postoperative complications such as stroke and necessitation of intra-aortic pump have been reported [12]. In another study, the role of WBC before CABG on increased rate of inpatient mortality and also mortality one year following the surgery was studied but the study did not evaluate the role of WBC on recurrent ischemia. 

The effect of neutrophils on myocardial function after MI and its effects on early postmyocardial infarction CHF have been studied [13]. Neutrophils and macrophages can cause myocardial reperfusion injury [14]. The effect of other acute phase reactants such as TNF, CRP and IL6 in prognosis of acute coronary syndromes is also known [15, 16]. The role of CRP, in atherosclerosis and its effects on patients' prognosis and mortality after CABG have been investigated [17–20]. The impact of elevated WBC before surgery on preoperative myocardial necrosis, inpatient mortality, and mortality one year after the surgery has also been shown. Probably, increased use of intra-aortic balloon pump during hospitalization is linked to elevated WBC count before surgery [11]. 

 Recently, in a large multicenter double-blind study, 1273 patients who underwent CABG were followed for up to11 years and the effect of hematologic elements and WBC in post-CABG prognosis was evaluated [12]. Increase WBC count was associated with poorer prognosis after CABG but this association was not related to the other parameters such as hematocrit or platelet count. There are some studies which have described the role of CRP in direct vascular injury as a preinflammatory and prothrombotic factor, mainly through activating tissue factors [13] and stimulation of TNF, IL6, and IL2 [21]. Overall, it is not clear whether higher levels of acute phase reactants are directly involved in vascular injury or they are just markers of poor prognosis after CABG and this is the subject of debate. 

Reduced inflammation-mediated organ damage as a consequence of leukocyte filtration has been reported [22].

Leukocyte filtration is based on reduction in active neutrophil contact with vascular endothelium and as a result a reduced inflammatory response. Studies have reported positive effects of leukocyte filtration in emergency surgeries [23, 24]. In contrast, Salamonsen et al. did not show any benefit of leukocyte reduction before elective CABG on decreasing early postsurgical complications, hospitalization duration, intubation period, and ICU stay. 

Our study also showed a correlation between BMI over 29 kg/m^2^ and the risk of restenosis one year after CABG surgery, when adjusted for other important variables. Obesity has been clearly established as an independent risk factor for the development of coronary artery disease [25, 26]. Romero-Corraland and his colleagues reported a comprehensive systematic review of all available cohort studies, includes 40 prospective studies with conflicting results. They found that the association between increased BMI and total mortality is significant only for sever obesity once coronary artery disease is established [27]. In spite of an unclear relationship between body weight and adverse outcome after CABG surgery, an overall review suggests that lowering body weight is the best preventive measure to decrease mortality after CABG.

## 7. Conclusion

This study adds more information to the pool of data about the role of inflammatory factors in cardiovascular mortality. It seems that elevated white blood cell count has a good association with the recurrent ischemic events after CABG. Probably patients with elevated WBC count, before CABG, would get more benefit from aggressive cardiovascular risk modification. 

## 8. Study Limitations

Although all samplings were done in five days before the surgery, sampling time was not the same in all patients. Therefore different sampling time might have some influence on our results. We did not check white blood cell count after surgery. We did not do angiography to find whether recurrent ischemic events were due to restenosis of grafted vessels or not. It was better that patients undergo angiography to determine cause of recurrent ischemia. Small population and short followup time of patients are another limitation. 

## Figures and Tables

**Table 1:** Base line characteristics of the studied population.

Total number	380
Male gender	281 (73.9%)
Mean age	57 ± 9.4

WBC count	
≥6000 mic/lit	253 (66.6%)
Hypertension	211 (55.5%)
Diabetes mellitus	49 (12.9%)
Smoker	162 (42.6%)
Family history	113 (29.7%)
BMI ≥ 29 kg/m^2^	103 (27%)

Cholesterol	
<200	234 (61.6%)
200–240	79 (20.6%)
>240	67 (17.6%)

Ejection fraction (%)	
<40	84 (22.1%)
40–50	158 (41.6%)
>50	138 (36.3%)
Left main > 50%	34 (8.9%)
LAD > 70%	342 (90%)
2VD	112 (29.5%)
3VD	171 (45%)
Cr > 1.5 mg/dL	18 (0.04%)
Off pump	205 (53.9%)
On pump	175 (46.1%)
Mean pump time	45 min

WBC: White Blood Cell; BMI: Body Mass Index; 2VD: 2 Vessel Disease.

**Table 2:** 30-day followup outcome in two WBC groups.

	WBC < 6000 (*n* = 127)	WBC ≥ 6000 (*n* = 253)	*P* value	OR (95% CI)
Atrial fibrillation	9 (16%)	39 (81.3%)	0.021	2.38 (1.1–5.1)
Myocardial infarction	8 (36.4%)	14 (63.6%)	0.76	0.87 (0.35–2.2)
Cardiac mortality	1 (25%)	3 (75%)	0.7	1.5 (0.15–1.4)
Unstable angina	0 (0%)	2 (100%)	0.31	0.6 (0.6–0.7)
Balloon pump	1 (33.3%)	4 (66.7%)	0.91	1.01 (0.09–11)

WBC: White Blood Cell; myocardial infarction: patients who met two of these three criteria were regarded to have MI: (1) chest pain prolonged more than 30 minutes, (2) ST elevation, and (3) increase in cardiac enzymes.

**Table 3:** One-year followup outcome in two WBC groups.

	WBC < 6000 (*n* = 127)	WBC ≥ 6000 (*n* = 253)	*P* value
Noncardiac mortality	2 (100%)	0 (0%)	0.045
Cardiac mortality	2 (22.2%)	7 (77.8%)	0.47
Stable angina	18 (22.8%)	61 (77.2%)	0.024
Unstable angina	5 (20%)	20 (80%)	0.14
Revascularization (PTCA or CABG)	0 (0%)	4 (100%)	0.15
Myocardial infarction	2 (28.6%)	5 (71.4%)	0.78
Total ischemic event	27 (22.7%)	92 (77.3%)	0.003

WBC: White Blood Cell. Stable angina: the patients who had chest pain with these three criteria: (1) exertional chest pain, (2) retrosternal, and (3) relived by sublingual Nitrate or rest; myocardial infarction: patients who met two of these three criteria were regarded to have MI: (1) chest pain prolonged more than 30 minutes, (2) ST elevation, and (3) increase in cardiac enzymes. Total ischemic event: patients who had one of this criteria: (1) stable angina, (2) unstable angina, (3) myocardial infarction, (4) cardiac mortality, and (5) revascularization (PTCA or CABG).

**Table 4:** Sensitivity, specificity, and positive and negative predictivevalues also area under the ROC curve for quantitative variable.

Variable	Sensitivity (%) (95% CI)	Specificity (%) (95% CI)	PPV (%) (95% CI)	NPV (%) (95% CI)	AUC ± SD, *P* Value
WBC					
≥6000 mic/lit	77 (67–84)	37 (31–43)	29 (24–35)	82 (74–88)	0.58 ± 0.033, *P* = 0.016
HTN	63 (54–72)	47 (41–54)	33 (27–40)	76 (69–83)	
Family history	32 (23–43)	71 (65–76)	28 (21–38)	74 (68–79)	
DM	16 (9–24)	88 (83–91)	32 (20–47)	74 (69–79)	
Smoking	48 (38–57)	59 (53–65)	32 (25–39)	74 (67–79)	
BMI ≥ 29 kg/m^2^	40 (30–50)	77 (72–82)	40 (31–50)	77 (71–81)	0.55 ± 0.035, *P* = 0.1

PPV: Positive Predictive Value; NPV: Negative Predictive Value; WBC: White Blood Cell; BMI: Body Mass Index.

**Table 5:** Univariate analysis.

	Ischemic event	No ischemic event	OR (95% CI)	*P* value
Sex (male)	83 (69.7%)	198 (75.9%)	1.36 (0.84–2.21)	0.208
Mean age	57.09 (±9.4)	57.7 (±9.4)		0.51
HTN	71 (59.7%)	140 (53.6%)	1.27 (0.82–1.98)	0.27
Smoker	52 (50.7%)	110 (42.1%)	1.065 (0.68–1.65)	0.77

Cholesterol				
200–240 mg/dL	27 (22.7%)	52 (19.9%)	1.16 (0.68–2)	0.57
>240 mg/dL	20 (16.8%)	47 (18%)	0.95 (0.52–1.73)	0.88
Diabetes Mellitus	16 (13.4%)	33 (12.6%)	1.07 (0.56–2.03)	0.82
BMI ≥ 29	42 (35.9%)	61 (23.7%)	1.79 (1.11–2.89)	0.015

WBC				
≥6000 mic/lit	92 (77.3%)	161 (61.7%)	2.11 (1.28–3.44)	0.003
Left main >50%	13 (10.9%)	21 (8%)	1.402 (0.67–2.9)	0.36
LAD > 70%	106 (89.1%)	236 (91%)	0.79 (0.38–1.62)	0.52

Ejection fraction				
40%–50%	51 (40.2%)	107 (42.3%)	0.84 (0.48–1.48)	0.55
>50%	49 (38.6%)	89 (32.2%)	0.83 (0.46–1.47)	0.52
Family history	33 (27.7%)	80 (30.7%)	0.86 (0.53–1.4)	0.56
Number of graft (mean)	2.5 (±0.91)	2.58 (±0.82)		0.38
Cr > 1.5 mg/dL	8 (6.7%)	10 (12.4%)	1.809 (0.69–4.7)	0.21
Pump timing (mean)/min	43.73 min	46.91 min		0.59
CPB (mean)/min	243/4 (±91)	243 (±86)		0.98
CK.MB	47 (±41)	43 (±25)		0.4
Logestic EuroScore	2.61 (±1.3)	2.2 (±1.2)		0.01
Arterial graft	85 (87.6%)	246 (86.9%)	1.06 (0.53–2.1)	0.85
ASA	94 (96.9%)	273 (96.5%)	1.1 (0.3–4.2)	0.83
Beta.blocher	79 (81.4%)	220 (77.3%)	1.2 (0.7–2.2)	0.41
Ace.Inh	46 (47.4%)	149 (52.7%)	0.81 (0.5–1.2)	0.37
CCB	11 (11.3%)	34 (12%)	0.93 (0.45–1.9)	0.85
Statin	61 (62.9%)	150 (0.53%)	1.5 (0.9–2.4)	0.09
Diuretic	6 (6.2%)	14 (4.9%)	1.27 (0.43–3.3)	0.63
Nitriglicerin	30 (30.9%)	72 (25.4%)	1.3 (0.79–2.1)	0.29

BMI: Body Mass Index; WBC: White Blood Cell; LAD: Left Anterior Descending; CCB: calcium chanel blocher.

**Table 6:** Multivariable analysis and adjusted odds ratio.

Variable	OR (before adjust)	OR (after adjust)	*P* value (after adjust)
WBC			
**≥**6000 mic/lit	2.11 (95% CI = 1.28–3.44)	2.25 (95% CI = 1.2–4)	0.005
Hypertension	1.27 (95% CI = 0.82–1.98)	1.17 (95% CI = 0.72–1.907)	0.54
Smoking	1.065 (95% CI = 0.68–1.65)	1.07 (95% CI = 0.64–1.7)	0.42
Diabetes mellitus	1.07 (95% CI = 0.56–2.03)	0.89 (95% CI = 0.39–1.6)	0.56
Age	0.99 (95% CI = 0.97–1.01)	0.97 (95% CI = 0.94–1.8)	0.83
BMI ≥ 29	1.79 (95% CI = 1.11–2.89)	2.007 (95% CI = 1.15–3.47)	0.013
Family history	0.86 (95% CI = 0.53–1.4)	1.05 (95% CI = 0.62–1.8)	0.82

Cholesterol			
200–240	1.16 (95% CI = 0.68–2)	0.8 (95% CI = 0.43–1.5)	
>240	0.95 (95% CI = 0.52–1.73)	1.01 (95% CI = 0.56–2.1)	0.79
Waist circumference	1.05 (95% CI = 0.68–1.62)	1.01 (95% CI = 0.58–1.7)	0.97
(Male > 102 cm and female > 88 cm)			
Cr > 1.5 mg/dL	1.809 (95% CI = 0.69–0.47)	0.95 (95% CI = 0.31–2.8)	0.93
Euro score	1.22 (95% CI = 1.03–1.4)	1.07 (95% CI = 1.1–1.6)	0.002
Arterial graft	0.97 (95% CI = 0.47–1.9)	1.07 (95% CI = 0.51–2.2)	0.84
CK.mb	0.91 (95% CI = 0.98–1.005)	0.99 (95% CI = 0.98–1.004)	0.28
